# DIGITAL INTERVENTIONS FOR PEOPLE WITH MILD COGNITIVE IMPAIRMENT: A SYSTEMATIC REVIEW OF RCTS

**DOI:** 10.1093/geroni/igae098.3313

**Published:** 2024-12-31

**Authors:** Chanchan He, Nan Jiang, Ke Chen, Yue Yu, Yexuan Xiao

**Affiliations:** Tsinghua University, Shenzhen, Beijing, China (People’s Republic); Tsinghua University, Beijing, Beijing, China (People’s Republic); Tsinghua University, Beijing, Beijing, China (People’s Republic); Tsinghua Shenzhen International Graduate School, Shenzhen, Guangdong, China (People’s Republic); Tsinghua University, Beijing, Beijing, China (People’s Republic)

## Abstract

**Background:**

Digital interventions aim to improve outcomes in irreversible Mild Cognitive Impairment (MCI) with limited evidence on effective strategies. This review evaluates the effectiveness of such interventions through randomized controlled trials (RCTs), identifying key characteristics of success.

**Methods:**

The search was conducted on CINAHL, Embase, MEDLINE, PubMed, PsycINFO, and grey literature from January 1, 2010, to November 20, 2023, to identify randomized controlled trials that examined the effects of digital interventions on global cognitive function outcomes in individuals with MCI. Mean differences and standard deviations of neuropsychological assessment scores were extracted to derive standardized mean differences.

**Results:**

Out of 2,859 studies, 19 were analyzed. Digital interventions improved global cognitive function in MCI patients (SMD (95% CI) = 0.40 (0.22-0.59)), executive function (-0.48 (-0.76 to -0.21)) and attention (0.39 (0.16-0.61)). Four critical factors contribute to the success of these interventions: (1) Ease of use; (2) Opportunities for social interactions; (3) Human support; (4) Engagement in physical exercises; and (5) interventions tailored to the participants’ needs. Additionally, methodological considerations for future randomized controlled trials on digital MCI interventions include: (1) Inclusion of both a healthy control group and an intervention group with clinically diagnosed MCI; (2) Collecting support-related data throughout the intervention period; (3) Assessing intervention success qualitatively and quantitatively; (4) Conducting follow-up interviews and surveys up to 1-year post-intervention to evaluate long-term outcomes.

**Conclusion:**

The insights from this systematic review empower future digital MCI intervention researchers, health professionals, clinicians, and patients to design, develop, and implement successful interventions for older users.

